# PDGFR-β-Positive Perivascular Adventitial Cells Expressing Nestin Contribute to Fibrotic Scar Formation in the Striatum of 3-NP Intoxicated Rats

**DOI:** 10.3389/fnmol.2018.00402

**Published:** 2018-11-05

**Authors:** Tae-Ryong Riew, Jeong-Heon Choi, Hong Lim Kim, Xuyan Jin, Mun-Yong Lee

**Affiliations:** ^1^Department of Anatomy, Catholic Neuroscience Institute, College of Medicine, The Catholic University of Korea, Seoul, South Korea; ^2^Integrative Research Support Center, Laboratory of Electron Microscope, College of Medicine, The Catholic University of Korea, Seoul, South Korea

**Keywords:** acute brain injury, brain macrophage, fibrotic scar, nestin, PDGFR-β, perivascular adventitial cell, reactive fibroblast, vimentin

## Abstract

Perivascular cells expressing platelet-derived growth factor receptor beta (PDGFR-β) have recently been implicated in fibrotic scar formation after acute brain injury, but their precise identity and detailed morphological characteristics remain elusive. This study sought to characterize and define the cellular phenotype of vascular-associated cells expressing PDGFR-β in the striatum of rats treated with the mitochondrial toxin 3-nitropropionic acid (3-NP). In the control striatum, PDGFR-β-positive cells were invariably localized on the abluminal side of smooth muscle cells of larger caliber vessels, and demonstrated morphological features typical of perivascular fibroblasts. PDGFR-β expression increased and expanded to almost all vessels, including microvessels in the lesion core, at 7 days after 3-NP injection. The cells expressing PDGFR-β had ultrastructural features of fibroblasts undergoing active collagen synthesis: large euchromatic nuclei with a prominent nucleolus, well-developed rough endoplasmic reticulum (rER) with dilated cisterns and extracellular collagen fibrils. By 14 days, PDGFR-β-positive cells had somata located at a distance from the vasculature, and their highly ramified, slender processes overlapped with those from other cells, thus forming a plexus of processes in the extravascular space of the lesion core. In addition, their ultrastructural morphology and spatial correlation with activated microglia/macrophages were elaborated by three-dimensional reconstruction. Using a correlative light- and electron-microscopy technique, we found that the intermediate filament proteins nestin and vimentin were induced in PDGFRβ-positive fibroblasts in the lesion core. Collectively, our data suggest that perivascular PDGFR-β-positive fibroblasts are distinct from other vascular cell types, including pericytes and contribute to fibrotic scar formation in the lesion core after acute brain injury. Nestin and vimentin play critical roles in the structural dynamics of these reactive fibroblasts.

## Introduction

Insults to the central nervous system (CNS), such as traumatic brain injury, spinal cord injury and ischemic strokes, trigger tissue scarring (for review, see Fernández-Klett and Priller, 2014). However, unlike those in other tissues, CNS scars can be categorized into two cytologically and biochemically distinct components: glial and fibrotic. While glial scars, characterized by hypertrophy and proliferation of astrocytes, have been extensively studied to date, fibrotic scars have garnered relatively less attention, until recently.

Fibrotic scars are generally considered more detrimental to the brain or spinal cord, as they impede axonal regeneration and function as a physical barrier that restricts inflammatory processes (Yoshioka et al., 2010; Cha et al., 2014; Zhu et al., 2015). This growing awareness of functional implications of fibrotic scars has generated particular interest in the cells that could produce these scars in response to CNS insults. Goritz et al. (2011) demonstrated that a subtype of pericytes, capillary-lining supportive perivascular cells, plays a key role in scar tissue formation after spinal cord injury. In their study, type A pericytes, a subpopulation of pericytes, detach from the blood vessels and become scar-forming fibrotic cells. In contrast, other studies have presented evidence that other types of vascular cells, perivascular stromal cells or perivascular fibroblasts, which are distinct from pericytes in their classical definition, contribute to fibrotic scar formation after CNS insults (Fernández-Klett et al., 2013; Soderblom et al., 2013). Despite the controversy, such cells, called by different names, share some characteristics, such as their perivascular localization and expression of a common marker, platelet-derived growth factor receptor beta (PDGFR-β). Since several cell types, including pericytes, perivascular macrophages and adventitial cells, reside within the perivascular space and there are no perfect markers for discriminating among them, their exact origin cannot be confirmed by light microscopic observation alone and requires immunoelectron microscopy studies.

Nestin, a type VI intermediate filament protein, was originally identified in multipotent CNS stem cells, and its role and expression in neural stem/progenitor cells in response to ischemic injury has been extensively investigated (Lendahl et al., 1990; Wiese et al., 2004; Park et al., 2010; Nakagomi et al., 2011; Nakata et al., 2017). Our previous study demonstrated that, in the ischemic core of stroke-lesioned rats, nestin expression is induced in a novel subset of vascular wall cells that are distinct from endothelial cells, pericytes, or smooth muscle cells. These cells could transform into fibroblast-like cells during the chronic phase (>14 days post-injury), suggesting that nestin plays a role in the structural remodeling of vascular cells (Shin et al., 2013). Therefore, we hypothesized that fibrotic scar-forming cells require the intermediate protein nestin in order to acquire their scar-forming phenotype after acute CNS injury.

In the present study, we first characterized the PDGFR-β-expressing cells that are thought to form fibrotic scars after brain injury induced by 3-nitropropionic acid (3-NP), an irreversible inhibitor of the mitochondrial respiratory chain complex II (Hamilton and Gould, 1987; Brouillet et al., 2005). This experimental model has advantages as a model of dynamic, spatiotemporal regulation of fibrotic scar formation in response to insults, because unlike ischemic stroke, 3-NP injection results in a well-demarcated striatal lesion that does not spread to other brain regions, such as the cerebral cortex, thus providing a better-preserved lesion core in an acute CNS injury model (Brouillet et al., 2005; Kim et al., 2015; Riew et al., 2017b). We provided detailed and precise information on their ultrastructural morphology using immunoperoxidase electron microscopy. Next, we showed that PDGFR-β/nestin double-positive vascular wall cells contribute to fibrotic scar formation and constitute a distinct population of vessel cells in terms of their morphology, and we describe the molecular characteristics of these cells by using correlative light- and electron microscopy.

## Materials and Methods

### Animal Preparation

All experimental procedures were conducted in accordance with the Laboratory Animal Welfare Act, the Guide for the Care and Use of Laboratory Animals, and Guidelines and Policies for Rodent Survival Surgery, and were approved by the Institutional Animal Care and Use Committee at the College of Medicine, The Catholic University of Korea (Approval Number: CUMC-2017-0321-02). All efforts were made to minimize animal suffering and to reduce the number of animals used.

Adult, male Sprague-Dawley rats (250–300 g, aged 9–11 weeks) were used in this study. Animals were housed in groups of three per cage in a controlled environment at a constant temperature (22 ± 5°C) and humidity (50 ± 10%) with food (gamma ray-sterilized diet) and water (autoclaved tap water) available *ad libitum*. They were maintained on a 12-h light/dark cycle. 3-NP (Sigma-Aldrich, St. Louis, MO, USA) was dissolved in buffered saline (pH 7.0), and administered intraperitoneally (i.p.) at a dose of 15 mg/kg once daily for 3 days. All 3-NP-injected rats were evaluated daily for the presence of behavioral deficits, and only rats exhibiting neurological deficit symptoms, including hind limb impairment or a kyphotic posture, recumbency and impaired postural adjustments, were included in the experimental group (Hamilton and Gould, 1987). Animals were sacrificed 3, 7, 14 and 28 days after the final injection of 3-NP (*n* = 6/time point). The control group (*n* = 3) received intraperitoneal injections of the same volume of normal saline for three consecutive days and were sacrificed 3 days after the final injection.

The animals were anesthetized with 10% chloral hydrate, sacrificed, and then perfused transcardially with 4% paraformaldehyde in 0.1 M phosphate buffer (PB; pH 7.4) The brain tissues were equilibrated with 30% sucrose in 0.1 M PB and frozen whole.

### Western Blot Analysis

For the immunoblot analysis, rats from four groups (controls, experimental rats at 3, 7 and 28 days after 3-NP injection) were perfused transcardially with 0.1 M PB under anesthesia (10% chloral hydrate; 4 mL/kg i.p.). The striatal tissues were carefully dissected under a stereoscopic microscope, and proteins were isolated from the striatum using lysis buffer (1% sodium dodecyl sulfate [SDS], 1.0 mM sodium orthovanadate, 10 mM Tris, pH 7.4). Equal amounts (20 μg) of total protein were separated by SDS-polyacrylamide gel electrophoresis (7.5%) and transferred to polyvinylidene difluoride membranes. Immunostaining of the blots was performed using the following primary antibodies: rabbit monoclonal antibody against PDGFR-β (1:1,000; Abcam, Cambridge, UK) and mouse monoclonal antibody against anti-β-actin (1:40,000; Sigma-Aldrich). Membranes were then incubated with peroxidase-coupled secondary antibodies (1:1,000; Millipore, Billerica, MA, USA) for 1 h at room temperature. Blots were developed using the Amersham ECL Prime western blotting detection reagent (GE Healthcare, Little Chalfont, UK). Samples from three animals were used for immunoblotting at each time point, and relative optical densities of the protein bands were obtained from three independent experiments, each performed in triplicate. Data were obtained by densitometry and were normalized using β-actin as the loading control.

### Immunohistochemistry

For PDGFR-β immunohistochemistry, coronal cryostat sections (25-μm-thick) were incubated overnight at 4°C with a rabbit polyclonal antibody against PDGFR-β (1:200; Abcam). Primary antibody binding was visualized using peroxidase-labeled goat anti-rabbit antibody (1:100; Jackson ImmunoResearch, West Grove, PA, USA) and 0.05% 3,3′-diaminobenzidine tetrahydrochloride (DAB) with 0.01% H_2_O_2_ as a substrate. The specificity of PDGFR-β immunoreactivity was confirmed by the absence of immunohistochemical staining in sections from which the primary or secondary antibody had been omitted. Tissue sections were scanned and photographed using a slide scanner (SCN400, Leica Microsystems Ltd., Mannheim, Germany). Images were converted to TIFF format, and contrast levels adjusted using Adobe Photoshop v. 13.0 (Adobe Systems, San Jose, CA, USA).

For the evaluation of tissue injury, serial sections from sham controls and experimental rats at 3 days post-lesion were processed for Fluoro-Jade B (FJB) histochemistry and for 32 kDa dopamine- and cyclic AMP-regulated phosphoprotein (DARPP-32) immunohistochemistry. For FJB staining, sections were stained with 0.0004% FJB (Millipore) in distilled water containing 0.01% acetic acid for 30 min according to the manufacturer’s protocol. After rinsing in distilled water, the sections were immersed in xylene and cover-slipped with DPX mounting medium (Sigma-Aldrich). For immunohistochemistry, sections were incubated at 4°C overnight with rabbit polyclonal antibody against DARPP-32 (1:200; Cell Signaling Technology, Danvers, MA, USA). Tissue sections were scanned and photographed using a slide scanner (Axio Scan.Z1, Carl Zeiss Co. Ltd., Oberkochen, Germany).

For triple-labeling, nonspecific staining was blocked by preincubation of free-floating sections (25-μm-thick) in blocking buffer (3% normal goat serum, 1% bovine serum albumin and 0.5% triton). Primary antibodies and dilutions were as follows: rabbit monoclonal antibody against PDGFR-β (1:200; Abcam), mouse monoclonal antibody against RECA1 (1:200; Bio-Rad, Hercules, CA, USA), chicken polyclonal antibody against glial fibrillary acidic protein (GFAP; 1:500; Millipore), goat polyclonal antibody against type IV collagen (1:100; Bio-Rad), mouse monoclonal antibody against nestin (1:500; Bio-Rad), goat polyclonal antibody against ionized calcium-binding adaptor molecule 1 (Iba1; 1:500; Abcam), chicken polyclonal antibody against vimentin (1:500, Millipore), mouse monoclonal antibody to NG2 (1:500; Millipore), or rabbit polyclonal antibody against Ki-67 (1:1,000; Leica Biosystems, Wetzlar, Germany). This was followed by a 2-h incubation with appropriate secondary antibodies, as follows: Cy3-conjugated donkey anti-goat antibody (1:2,000; Jackson ImmunoResearch), Cy3-conjugated donkey anti-mouse antibody (1:2,000; Jackson ImmunoResearch), Cy3-conjugated goat anti-mouse antibody (1:2,000; Jackson ImmunoResearch), Cy3-conjugated goat anti-rabbit antibody (1:2,000; Jackson ImmunoResearch), Alexa Fluor 488-tagged donkey anti-rabbit antibody (1:300; Thermo Fisher, Waltham, MA, USA), Alexa Fluor 488-tagged goat anti-rabbit antibody (1:300; Thermo Fisher), Alexa Fluor 488-tagged goat anti-mouse antibody (1:300; Thermo Fisher), Cy5-conjugated donkey anti-goat antibody (1:1,000, Jackson ImmunoResearch), Alexa Fluor 647-tagged goat anti-chicken antibody (1:500; Abcam), or Alexa Fluor 647-tagged donkey anti-mouse antibody (1:300; Thermo Fisher). Negative staining controls for triple-immunofluorescence involved omission of the primary or secondary antibodies. In addition, we compared the results of triple-labeling with those from single- and double-labeling of all combinations of antibodies to ensure clear interpretation of results. Counterstaining of cell nuclei was carried out using DAPI (4′,6-diamidino-2′-phenylindole; 1:2,000; Roche, Mannheim, Germany) for 10 min.

In addition, double-labeling was performed using a mixture of a rabbit polyclonal antibody against PDGFR-β (1:200; Abcam), and one of the following antibodies: mouse monoclonal antibody to NG2 (1:500; Millipore) or goat polyclonal antibody to α-smooth muscle actin (α-SMA; 1:100; Bio-Rad). Some sections were also incubated at 4°C overnight with a mixture of mouse monoclonal antibody against nestin (1:500; Bio-Rad) and either rabbit polyclonal antibody against laminin (1:100; Sigma-Aldrich) or fibronectin (1:300; Sigma-Aldrich).

In order to label proliferating cells, rats were intraperitoneally injected with 5-bromo-2′-deoxyuridine (BrdU; Sigma-Aldrich, 50 mg/kg) once daily on days 1 and 2 before being sacrificed on day 3 post-lesion. For triple-immunofluorescence histochemistry for BrdU, sections were pretreated to denature DNA and were then incubated with a rat monoclonal anti-BrdU antibody (1:200; Accurate Chemical and Scientific Corporation; Westbury, NY, USA), rabbit monoclonal anti-PDGFR-β (1:200; Abcam), and mouse monoclonal anti-nestin (1:500; Millipore). The sections were then incubated in a mixture of Cy3-conjugated donkey anti-mouse antibody (1:2,000; Jackson ImmunoResearch), Alexa Fluor 488-tagged goat anti-rabbit antibody (1:300; Thermo Fisher) and Cy5-conjugated goat anti-rat antibody (1:500; Abcam) for 2 h at room temperature.

Slides were viewed under a confocal microscope (LSM 700; Carl Zeiss Co. Ltd., Oberkochen, Germany) equipped with four lasers (Diode 405, Argon 488, HeNe 555 and HeNe 639) under constant viewing conditions. Images were converted to TIFF format, and contrast levels and sizes were adjusted using Adobe Photoshop v.13.0.

### Quantitative Analysis of Vascular PDGFR-β Expression and the Size of the Lesion Core

We assessed the relationship between the intensity of PDGFR-β immunoreactivity and the vessel diameter. Five randomly selected areas (160 × 160 μm per field) were chosen in the control striatum (*n* = 3 animals), and the maximum diameter of the vessel was measured in randomly selected blood vessels. The vessels were divided into two groups: capillary-like microvessels (<7.5 μm in diameter) and larger vessels (>8.5 μm in diameter), according to the vessel diameter, and the intensity of PDGFR-β immunoreactivity was measured in each group.

In addition, to quantify time-dependent changes in PDGFR-β expression associated with the vasculature after 3-NP injection, we measured the area immunostained with PDGFR-β among all RECA-1-positive vascular structures in the controls and in experimental rats at days 3 and 7 post-lesion. Three coronal sections per animal (*n* = 3 animals per time point) were taken from the invariable region 0.20–1.20 mm anterior to the bregma (Paxinos and Watson, 2006). Five randomly selected areas (160 × 160 μm per field) were chosen in the lesion core of each section, and images of PDGFR-β and RECA-1 immunoreactivity were then obtained from these areas at ×400 magnification under constant viewing conditions. The area covered by PDGFR-β and RECA-1 was estimated with Zen 2.3 blue edition (Carl Zeiss Co. Ltd.). The coverage of PDGFR-β was expressed as a percentage of the total RECA-1-positive vascular area. We also measured the areas covered by PDGFR-β in the extravascular spaces that were devoid of RECA-1 immunoreactivity. Sections double-labeled for PDGFR-β and RECA1 were obtained from controls and experimental rats at 3, 7, 14 and 28 days post-lesion. Quantification was performed as described above. Intensity profiles of specific areas were obtained using Zen 2010 (Carl Zeiss Co. Ltd.).

To assess the evolution in the size of the lesion core, the maximum horizontal distance of GFAP-negative striatal area was quantified from three to five coronal tissue sections labeled with GFAP from controls and experimental rats at 3, 7, 14 and 28 days post-lesion (*n* = 5 animals per time point) as described above; all these images were obtained with a slide scanner (Axio Scan.Z1, Carl Zeiss Co. Ltd.).

Selecting animals, tissue sections, and certain regions of interests, and quantification were conducted in a randomized and blinded manner.

### Quantitative Analysis of the Microglia/Macrophages

To count the number of Iba1-positive microglia/macrophages and microglia/macrophages harboring contracts with PDGFR-β in the lesion core and peri-lesional area at day 7 post-lesion, 3D surface rendering of Iba1-positive microglia/macrophages was first performed using IMARIS software (Bitplane, Zurich, Switzerland) and then this rendering was used to count the contacting Iba1-positive cells. Three coronal sections (*n* = 3 animals), double-labeled for PDGFR-β and Iba1, were taken from the invariable region 0.20–1.20 mm anterior to the bregma (Paxinos and Watson, 2006) and five randomly selected areas (80 × 80 μm per field) were chosen in the lesion core and peri-lesional area of each section, and images of PDGFR-β and Iba1 immunoreactivity were then obtained from these areas at ×400 magnification under constant viewing conditions. Iba1-positive cells were scored only when their nuclei could be clearly seen, and among them, cells clearly contacting PDGFR-β-positive cells were counted, separately. Analyses were conducted in a blinded and randomized fashion.

### Immunoelectron Microscopy

For pre-embedding immunoelectron microscopy, floating vibratome sections (50-μm-thick) from control and experimental rats 3, 7, 14 and 28 days post-lesion were immunostained with a rabbit monoclonal antibody to PDGFR-β (1:200; Abcam). Immunoreactions were visualized using DAB as a chromogen. After post-fixation, dehydration and embedding in Epon 812, areas of interest were excised and glued onto resin blocks. After being cut into ultrathin sections (70–90-nm-thick), they were observed under an electron microscope (JEM 1010, JEOL, Tokyo, Japan) with slight uranyl acetate staining.

For the correlative light- and electron-microscopic study, vibratome sections (300-μm-thick) from experimental rats at 7 and 14 days after 3-NP injection were cryoprotected with 2.3 M sucrose in 0.1 M phosphate buffer and frozen in liquid nitrogen. Semi-thin cryosections (2-μm-thick) were cut at −100°C with a glass knife in a Leica EM UC7 ultramicrotome equipped with an FC7 cryochamber (Leica). The sections were double-labeled using a rabbit monoclonal antibody to PDGFR-β (1:200; Abcam) and mouse monoclonal antibody to nestin (1:500; Millipore). Antibody staining was visualized using Cy3-conjugated goat anti-mouse antibody (1:2,000; Jackson ImmunoResearch) and Alexa Fluor 488-tagged goat anti-rabbit antibody (1:300; Thermo Fisher). Sections were labeled with DAPI for 10 min. Coverslipped sections were examined with a confocal microscope and photographed at various magnifications with a differential interference contrast setting to find specific areas for later examination by electron microscopy. After the coverslips had been floated off the sections, the tissues were prepared further for electron microscopy, as described previously (Riew et al., 2017a).

### Three-Dimensional Reconstruction

For 3D reconstruction of immuno-electron microscopy, 25–30 consecutive serial sections (90-nm-thick) of Epon-embedded DAB-labeled slices were obtained from rats at 14 days after 3-NP injection. Two successive sections were aligned via rotation and translation, such that corresponding structures in the two sections were superimposed. To compensate for distortions introduced by sectioning, we transformed the images anisotropically and linearly. Alignment was followed by contouring the nucleus and cell membranes of PDGFR-β-positive cells, activated microglia/macrophages, and endothelial cells, by manually tracing these in different colors using Adobe Photoshop. Then, three-dimensionally reconstructed images were made from the stacks of all contoured sections using the 3D modeling program Mimics 10.0 (Materialise, Leuven, Belgium).

### Statistical Methods

Statistical significance was determined by Student’s *t*-test or one-way analysis of variance (ANOVA) followed by Tukey’s multiple comparisons test. Differences with *P* values of less than 0.05 were considered statistically significant. All statistical calculations were performed using GraphPad Prism version 5 (GraphPad Software Inc., San Diego, CA, USA). The exact *P* values are indicated on graphs and text.

Power analysis were performed using StudySize 3.0 (CreoStat HB, Sweden). The group size, and number of blood vessels, tissue sections and Iba1+ cells were determined to provide at least 80% power with a probability of type I error (α) = 0.05, an effect size of 0.15 for western blot analysis, mean intensity of 20 and change of 20% or 2,000 μm^2^ for PDGFR-β expression, 0.5 mm for lesion size, and 5 cells or a ratio of 0.15 for Iba1+ cell counts.

## Results

### Expression of PDGFR-β Is Increased in the Striatum of Rats Subjected to 3-NP Injection

**Figure 1:**
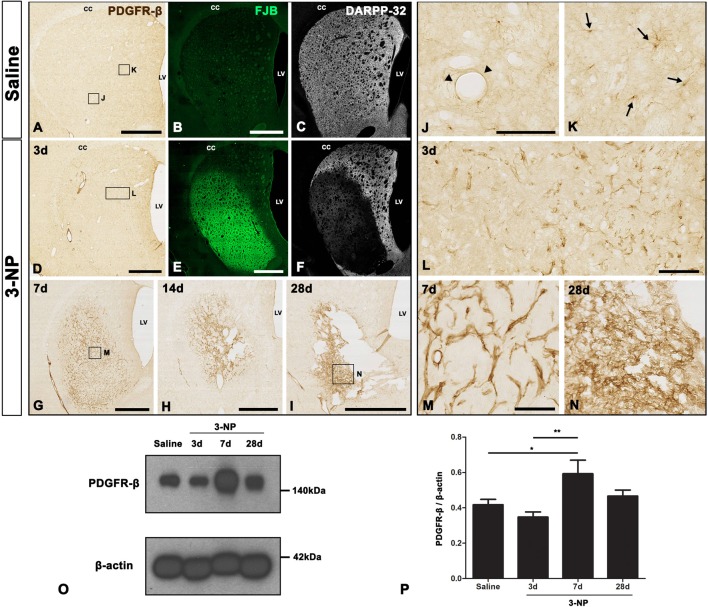
Temporal expression of platelet-derived growth factor receptor beta (PDGFR-β) in control and lesioned striatum after 3-nitropropionic acid (3-NP) injection. **(A–N)** Lower- **(A–I)** and higher- **(J–N)** magnification views of representative coronal sections from saline-treated control rats and rats subjected to 3-NP treatment. **(A–C,J,K)** Weak immunoreactivity for PDGFR-β is observed in the control striatum, where no specific staining for Fluoro-Jade B (FJB) is seen, but 32 kDa dopamine- and cyclic AMP-regulated phosphoprotein (DARPP-32)-positive striatal neurons are prominent. Higher magnification images reveal PDGFR-β expression in vascular profiles (arrowheads in **J**) and cells with thin and ramified processes (arrows in **K**). **(D–F,L)** On day 3 post-lesion, vessel-associated PDGFR-β immunoreactivity is evident in the core of a lesion in the lateral part of the striatum, which is clearly distinguished by intense FJB staining and concomitant loss of DARPP-32-positive neurons. **(D–I,M,N)** At days 7 **(G,M)**, 14 **(H)** and 28 **(I,N)**, PDGFR-β expression seems to become more densely distributed in the lesion core over time. At 28 days, PDGFR-β expression appears to form a network in the lesion core, and shrinkage of the striatum is prominent. CC, corpus callosum; LV, lateral ventricle. **(O)** Representative immunoblot analysis for PDGFR-β in microdissected striatum of saline-treated controls (Saline) and 3-NP-treated rats at 3, 7 and 28 days post-lesion. A band is present at 140 kDa, corresponding to the PDGFR-β protein. **(P)** Quantification of PDGFR-β protein expression. Data are obtained by densitometry and are normalized using β-actin as the loading control. The intensity of PDGFR-β protein expression in the lesioned striatum significantly increases at 7 days after 3-NP administration, and then declines, although enhanced expression levels persist until at least day 28. Relative optical densities of the protein bands were measured in triplicate in each of three animals at each time point. Data are expressed as mean ± SEM. *P < 0.05; **P < 0.01 vs. saline-injected controls and the day 3 data. Scale bars = 1 mm for **(A–I)**; 100 μm for **(J–N)**.

We analyzed the spatiotemporal dynamics of PDGFR-β expression in 3-NP-treated rats using immunohistochemistry. We observed no immunoreactivity specific to PDGFR-β when the primary or secondary antibody was omitted (data not shown). Figures 1A–I,J–N, representative higher- and lower-magnification images, respectively, show reproducible time-dependent changes in the expression of PDGFR-β in the striata of rats subjected to 3-NP injections. In the striata of saline-treated controls, where we observed no specific staining for FJB, but found evident DARPP-32-positive striatal neurons, weak PDGFR-β expression was observed in two cell types that differed according to their morphology and topographical distribution. These included cells associated with medium to large vasculature, as well as cells with ramified processes resembling NG2-glia, as previously reported (Kyyriäinen et al., 2017). Three days after the last 3-NP injection, intense PDGFR-β immunoreactivity was noted on the vascular profiles of varying sizes in the lesion core, which had been defined by strong FJB staining and concomitant loss of DARPP-32-positive neurons. At days 7, 14 and 28 post-lesion, PDGFR-β immunoreactivity had increased in the lesion core over time, forming a web-like network at day 28, when shrinkage of the striatum was prominent.

The expression profile of PDGFR-β was further examined by western blot analysis of protein extracted from the striatum of controls and experimental rats. Immunoblotting revealed that one major band of about 140 kDa, corresponding to PDGFR-β, had increased by 7 days after 3-NP administration, and these enhanced expression levels were maintained until at least day 28 (Figures 1O,P).

### Characterization of PDGFR-β-Positive Cells in the Control Striatum

**Figure 2:**
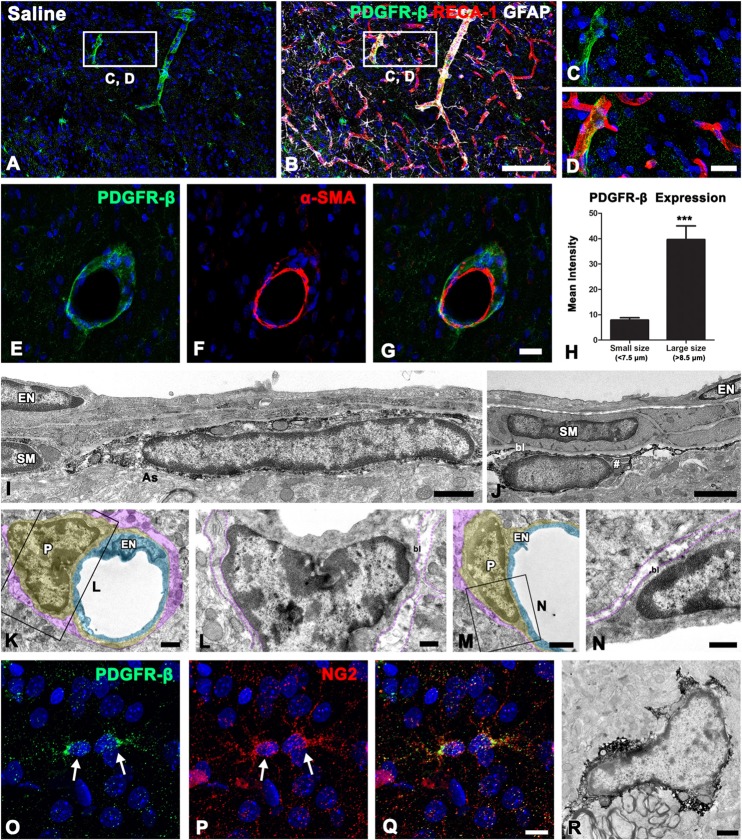
PDGFR-β expression profiles and their spatial relationship with vasculature in the control striatum. **(A–D)** Triple-labeling for PDGFR-β, glial fibrillary acidic protein (GFAP), and the endothelial cell marker RECA-1 in the control striatum, showing that PDGFR-β expression is localized in vascular profiles with a larger caliber, but not in capillary-like microvessels. The boxed areas in **(A,B)** are enlarged in **(C,D)**, respectively. **(E–G)** Double-labeling for PDGFR-β and the smooth muscle cell marker α–smooth muscle actin (α–SMA), showing that PDGFR-β expression does not colocalize with α–SMA, but is localized along the outer part of smooth muscle cells. **(H)** Quantitative analysis of the intensity of PDGFR-β immunoreactivity over the microvessels (<7.5 μm) and larger vessels (>8.5 μm) in the control striatum. Note that the intensity of PDGFR-β immunoreactivity in larger vessels is significantly higher than that in the capillaries. The data are expressed as the mean ± SEM. ***P < 0.001 vs. microvessels. **(I–N)** Ultrastructural analysis of PDGFR-β-positive cells associated with the venule **(I)** and arteriole **(J)** in the control striatum. PDGFR-β-positive cells have elongated or flattened nuclei and thin processes that invariably lie between the smooth muscle (SM) cells and the glia limitans (as in **A**), containing intermediate filaments (arrow in **I**) in venules **(I)** and arterioles **(J)**. Note that smooth muscle cells and endothelial cells (EN) are devoid of PDGFR-β. **(K–N)** No specific PDGFR-β immunoreactivity can be detected in capillaries that consist of a single layer of (EN; light blue) and pericytes (P; yellow). Note that the basal lamina (bl) surrounding pericytes is in direct contract with the glia limitans (magenta). **(O–Q)** Double-labeling for PDGFR-β and NG2, showing that both are colocalized in small stellate cells with fine processes (arrowheads). **(R)** Parenchymal PDGFR-β-positive cells have scarce cytoplasmic organelles and several processes. Cell nuclei are stained with DAPI. Scale bars = 100 μm for **(A,B)**; 20 μm for **(C–G)**; 10 μm for **(O–Q)**; 1 μm for **(I–K,M,R)**; 0.5 μm for **(L,N)**.

As described above, PDGFR-β immunoreactivity appeared to be associated with the vasculature in control striata. To clarify the phenotype of PDGFR-β-positive cells associated with the vasculature, we performed triple-labeling for PDGFR-β, the vascular endothelial cell marker RECA-1, and GFAP. In controls, PDGFR-β expression was localized within RECA-1-positive vascular profiles of a larger caliber, but capillary-like microvessels were devoid of PDGFR-β (Figures 2A–D). Double-labeling for PDGFR-β and the Smooth muscle cell marker α-SMA revealed that PDGFR-β expression was detected exclusively in α-SMA-positive larger caliber vessels and was localized along the outer part of smooth muscle cells (Figures 2E–G). To support the differential expression of PDGFR-β according to the vessel size, the relationship between the vessel diameter and the intensity of PDGFR-β immunoreactivity in the control striata was analyzed. The blood vessels were divided into capillary-like microvessels (<7.5 μm) and larger vessels (>8.5 μm) by their maximum diameter. Mean capillary diameter was 6.76 ± 0.68 μm in the control striata and the mean diameter of larger vessels was 15.48 ± 6.94 μm. The resulting data revealed that the intensity of PDGFR-β immunoreactivity in larger vessels was significantly greater than that in capillaries (Figure 2H).

To clarify the cellular identity of vessel-associated PDGFR-β-positive cells further, pre-embedding immunoelectron microscopy was performed. In the control striata, PDGFR-β-positive cells were invariably associated with larger caliber vessels (arterioles and venules), possessing smooth muscle cells. These cells had elongated or flattened nuclei with thin peripheral heterochromatin, and gave rise to thin processes located on the abluminal side of endothelial cells and smooth muscle cells, both of which were negative for PDGFR-β (Figures 2I,J). PDGFR-β-positive cells closely abutted the glia limitans, which was composed of astroglial processes containing intermediate filaments (Figures 2I,J). However, no specific PDGFR-β immunoreactivity was detected in capillaries that consisted of a single layer of endothelial cells and pericytes (Figures 2K–N). In addition to perivascular PDGFR-β-positive cells, PDGFR-β expression was detected in small stellate cells with fine processes (Figure 2O), as described above. These parenchymal PDGFR-β-positive cells coexpressed NG2 (Figures 2P,Q), as reported previously (Garbelli et al., 2015). Immunoelectron microscopic observations of parenchymal PDGFR-β-positive cells revealed that they had scarce cytoplasmic organelles and had several processes (Figure 2R).

### PDGFR-β Expression Is Associated With the Vasculature in the Lesion Core in the Early Phase After 3-NP Injection

**Figure 3:**
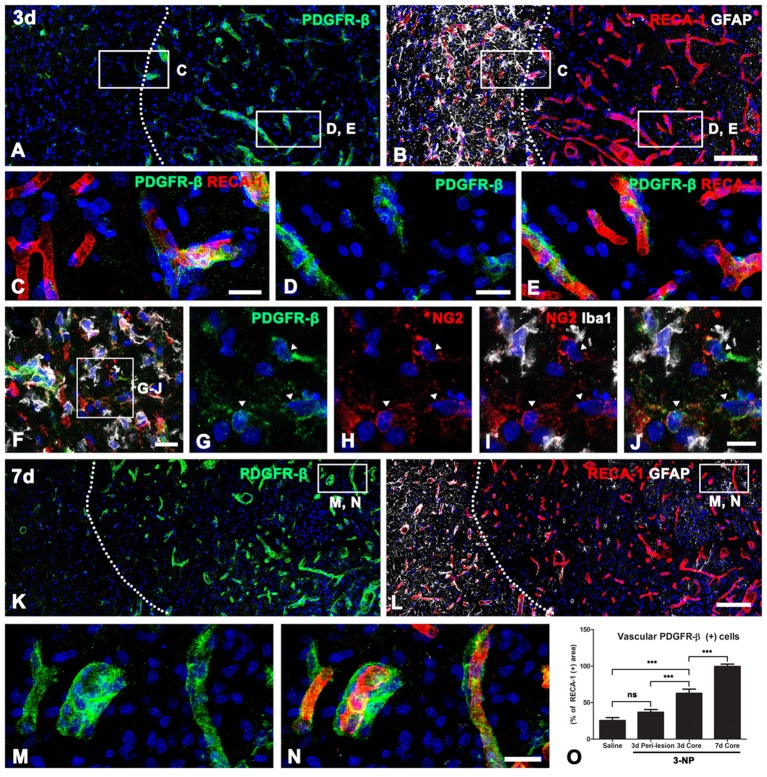
PDGFR-β expression profiles and their spatial relationship with vasculature in lesioned striatum at days 3 and 7 after 3-NP injection. **(A,B)** Triple-labeling for PDGFR-β, GFAP and the endothelial cell marker RECA-1 at 3 days post-lesion, showing that there is a marked increase in PDGFR-β expression confined to the vasculature in the lesion core, where GFAP immunoreactivity is absent. The broken line indicates the border between the lesion core and the peri-lesional area. **(C–E)** Higher magnification views of the peri-lesional area **(C)** and lesion core **(D,E)** in **(A,B)**. In the peri-lesional area (left half in **C**), PDGFR-β immunoreactivity is observed only in a small fraction of vessels. **(D–E)** In contrast, intense PDGFR-β expression is associated with most of the vasculature, including microvessels in the lesion core. **(F–J)** Triple-labeling of PDGFR-β, NG2 and Iba1, showing that parenchymal PDGFR-β-positive cells can be still detected in the extravascular area within the lesion core, where they are labeled with NG2, but not with Iba1. **(K–N)** At 7 days post-lesion, PDGFR-β immunoreactivity is localized in or in close vicinity to nearly all vessels in the lesion core (right side of the broken line). **(M,N)** Higher magnification views of the boxed areas in **(K,L)**, respectively. **(N)** The proportion of vascular areas occupied by PDGFR-β immunoreactivity within all RECA1-positive vessels. **(O)** Note that PDGFR-β increases significantly in the lesion core at 3 and 7 days post-lesion. The data are expressed as the mean ± SEM. ***P < 0.001 vs. saline-treated controls. Cell nuclei are stained with DAPI. ns: non-specific. Scale bars = 100 μm for **(A,B,K,L)**; 20 μm for **(C–F,M,N)**; 10 μm for **(G–J)**.

We next characterized PDGFR-β-positive cells in the lesion core by triple-labeling for PDGFR-β, RECA-1 and GFAP. At 3 days post-lesion, intense PDGFR-β expression was associated with most of the vasculature, including microvessels, in the lesion core, which was clearly demarcated by the absence of GFAP immunoreactivity, as described previously (Choi et al., 2017; Figures 3A,B,D,E). In contrast, PDGFR-β immunoreactivity in the peri-lesional area, which was characterized by the presence of astrocytes with reactive phenotype, was observed only in a small fraction of the vasculature (Figures 3A–C). Next. we performed triple-labeling of PDGFR-β, NG2 and Iba1 to determine whether parenchymal PDGFR-β-positive cells could be related to increased vascular PDGFR-β expression. As shown in Figures 3F–J, parenchymal PDGFR-β-positive cells could still be detected in the extravascular area within the lesion core, where they were labeled with NG2, but not with Iba1 antibodies (Figures 3F–J). Seven days post-lesion, PDGFR-β immunoreactivity was localized to or in close vicinity to nearly all vessels in the lesion core (Figures 3K–N).

Next, we determined the relative proportion of vascular profiles occupied by PDGFR-β among all RECA1-positive vessels in the lesion core, at days 3 and 7 post-lesion. As shown in Figure 3O, PDGFR-β expression covered only a small part (25.5%) of all RECA-1-positive vessels in controls rat striata. At 3 days post-lesion, this proportion rose to 62.8% in the lesion core, which was significantly higher than that in the peri-lesional area (36.8% of all vessels). At 7 days post-lesion, the PDGFR-β-positive area comprised 99.6% of the vascular area in the lesion core.

**Figure 4:**
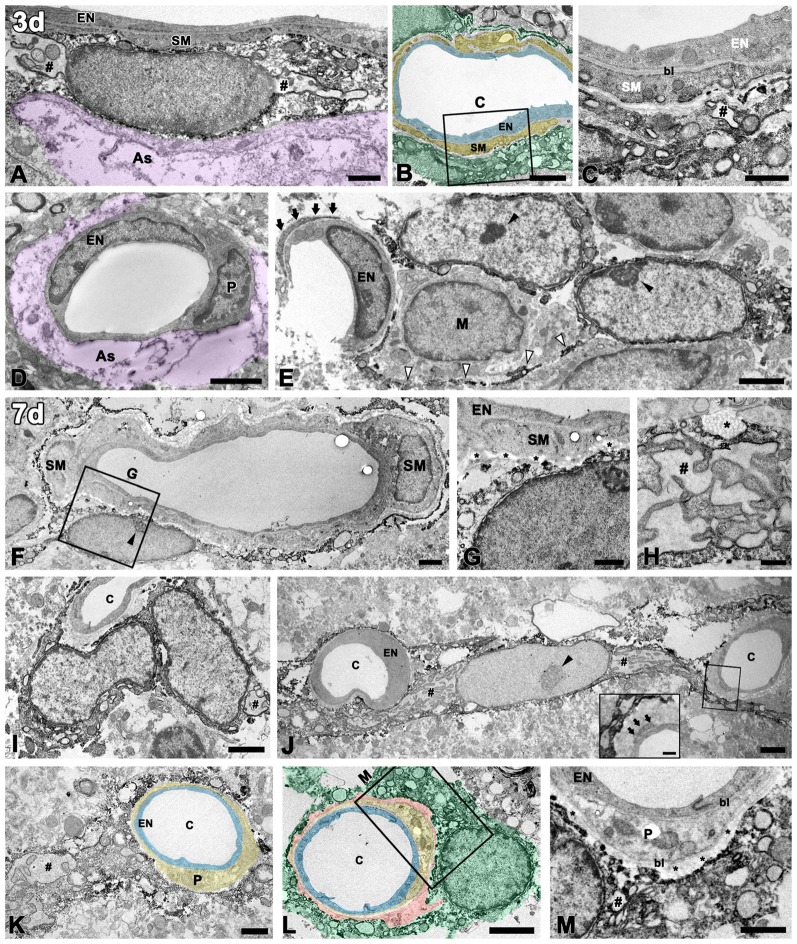
Ultrastructural analysis of PDGFR-β-positive cells associated with the vasculature in the lesioned striatum at days 3 and 7 after 3-NP injection. **(A–C)** At 3 days post-lesion, PDGFR-β-positive cells are characterized by large euchromatic nuclei and cytoplasmic distention, with dilated cisternae of rough endoplasmic reticulum (rER, #). Note that PDGFR-β-positive cells are located on the abluminal side of smooth muscle cells (SM) in venules **(A)** and arterioles **(B,C)**. **(C)** A higher magnification image of the boxed area in **(B)**, showing that PDGFR-β-positive processes form a multilayered sheath that covers the smooth muscle (SM) cells completely. **(D)** PDGFR-β immunoreactivity is not detected in endothelial cells (EN) and pericytes (P) of capillaries. The magenta-colored structures (as in **A,D**) indicate the degenerating glial limitans. **(E)** PDGFR-β-positive cells that have large euchromatic nuclei with prominent nucleoli (close arrowheads) extend long cytoplasmic processes (open arrowheads) that are apposed to the capillaries. Arrows indicate the pericyte processes. M, macrophage. **(F,G)** At day 7, PDGFR-β-positive cells are localized on the abluminal side of a smooth muscle (SM) cell and endothelial cells (EN). **(G)** A higher magnification image of the boxed area in **(F)**, showing that PDGFR-β-positive processes are closely associated with bundles of collagen fibrils (asterisks in **G**). **(H)** A part of a PDGFR-β-positive cell with a dilated rER showing a membrane-bound granule with filamentous contents (asterisk). **(I–M)** PDGFR-β-positive cells and processes appear to wrap around or directly abut the abluminal surface of endothelial cells (EN) and pericytes (P) of capillaries (c). Note that PDGFR-β-positive cells have large euchromatic nuclei with prominent nucleoli (arrowhead in **G**) and well-developed rER. (inset in **J,M**) Higher magnification images of the boxed areas in **(J,L)**, respectively, showing that PDGFR-β-positive cells and processes are clearly segregated by the bl of the pericytes that envelop the endothelial cells. Note that PDGFR-β-positive cells are closely associated with bundles of collagen fibrils (asterisks in **M**). Light blue indicates endothelium in **(B,K,L)**, yellow in **(B,K,L)** indicates the smooth muscle cell layer **(B)** and pericytes **(K,L)**, respectively; orange indicates collagen fibril deposition in **(L)**, and green indicates PDGFR-β-positive cells in **(B,L)**. # in **(H,I,J,K,M)** indicate the dilated rER with dilated interconnecting cisternae. Scale bars = 2 μm for **(B,D–F,I,J,L)**; 1 μm for **(A,C,G,K,M)**; 0.5 μm for **(H)**.

We further characterized the vessel-associated PDGFR-β-positive cells in the lesion core by immunoelectron microscopy. At 3 days post-lesion, when a degenerating glial limitans was evident in the lesion core, PDGFR-β-expressing cells in the 3-NP-treated rats could be distinguished from those in the controls by their larger euchromatic nuclei and cytoplasmic distention with dilated cisternae of rough endoplasmic reticulum (rER), but they were still located on the abluminal side of smooth muscle cells (Figure 4A). In addition, PDGFR-β-positive cell bodies and processes formed a multilayered sheath that was apposed to smooth muscle cells (Figures 4B,C). In capillaries, PDGFR-β immunoreactivity was not detected in the endothelial cells and pericytes of capillaries (Figure 4C), but some PDGFR-β-positive cells had long cytoplasmic processes that were apposed to the capillaries (Figure 4E).

At day 7, PDGFR-β-positive cells that had large euchromatic nuclei with prominent nucleoli were still localized along the outer part of smooth muscle cells (Figures 4F,G). In particular, they were frequently surrounded by collagen fibrils, which were marked in the perivascular space (Figure 4G). Interestingly, profiles of membrane-bound granules with apparent filamentous contents, suggestive of collagen, were present along the surface of PDGFR-β-positive cells with dilated rERs (Figure 4H). At this time point, PDGFR-β-positive cells were frequently found in close proximity to the capillaries, and their processes often wrapped or extended beyond the capillaries, or occasionally directly abutted the abluminal surface of the endothelial cells or pericytes (Figures 4I–M). In particular, higher magnification images clearly revealed that PDGFR-β-positive cells and processes were clearly segregated by the basal lamina of the pericytes that enveloped the endothelial cells (Figures 4J inset, 4K,M). In particular, these cells had a well-developed rER with interconnecting cisternae, some of which showed marked dilatation (Figures 4H,J,K).

### PDGFR-β Expression Increases in the Extravascular Area Over Time After 3-NP Injection and Is Closely Associated With Collagen Fibers in the Lesion Core

**Figure 5:**
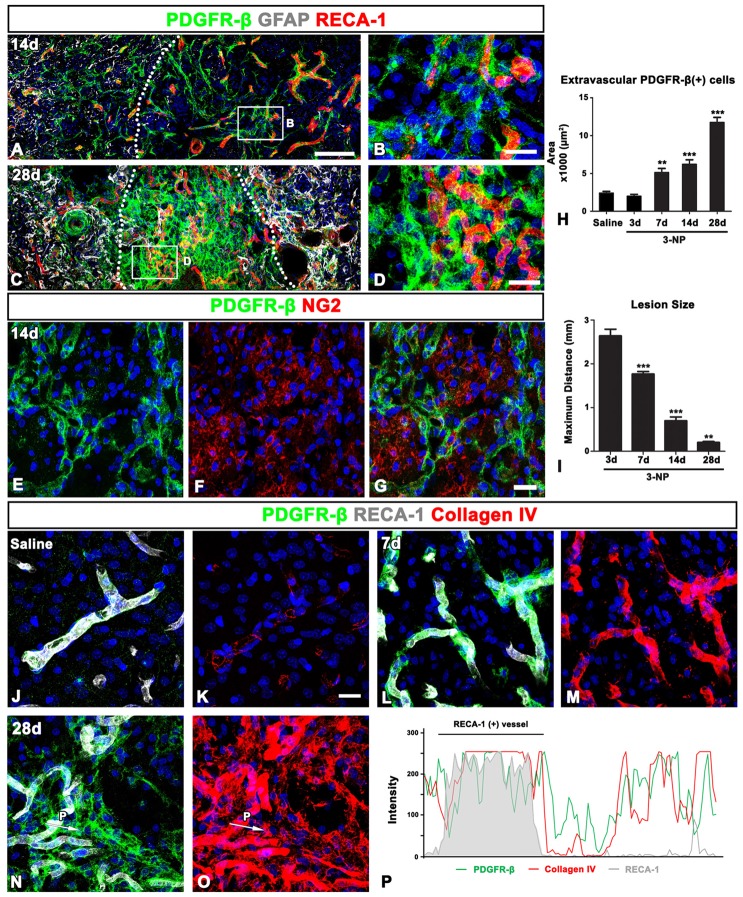
PDGFR-β expression profiles and their spatial relationship with vasculature and collagen in the control and lesioned striatum. **(A–D)** Triple-labeling for PDGFR-β, GFAP and the endothelial cell marker RECA1 at days 14 **(A,B)** and 28 **(C,D)**, showing that PDGFR-β expression is present in both vascular profiles and the surrounding extravascular neural tissue in the lesion core. Note the more-prominent enrichment of PDGFR-β expression in extravascular areas at 28 days. The broken lines indicate the border between the peri-lesional area and the lesion core, in which GFAP immunoreactivity is absent. **(B,D)** Higher magnification images of the boxed areas in **(A,C)**, respectively. **(E–G)** Double-labeling for PDGFR-β and NG2, showing that the extravascular network, mainly composed of PDGFR-β-positive processes, is devoid of NG2 expression. **(H)** Quantitative temporal analysis showing that the extravascular area occupied by PDGFR-β expression in the lesion core increases significantly until 28 days. **(I)** Quantitative temporal analysis showing the progressive decrease in the maximum horizontal distance of the lesion core over time. The data are expressed as the mean ± SEM. **P < 0.01, ***P < 0.001 vs. saline-treated controls or the day 3 data, respectively. **(J–O)** Triple-labeling for PDGFR-β, RECA-1 and collagen IV in the control **(J,K)** and lesioned striatum at days 7 **(L,M)** and 28 **(N,O)** post-lesion. PDGFR-β-positive vessels in the controls show very weak immunoreactivity for collagen IV, but they reveal intense collagen expression at 7 days post-lesion. At 28 days, a close relationship between PDGFR-β and collagen IV can be observed in the lesion core, where both are observed in vascular and extravascular areas. **(P)** Histogram of the intensity profiles of PDGFR-β-positive and collagen IV-positive signals along the indicated area (white arrows in **N,O**). Note that the two signals share overlapping spatial profiles within vascular (RECA-1-positive) and extravascular areas. Cell nuclei are stained with DAPI. Scale bars = 100 μm for **(A,C)**; 20 μm for **(B,D,E–G,J–O)**.

In the lesion core at 14 days post-lesion, PDGFR-β expression was still associated with vessels, but was also distributed in the surrounding extravascular neural tissue (Figures 5A,B). In particular, PDGFR-β-positive cell somata and processes in the extravascular areas were frequently closely apposed or intermingled with one another, forming a web-like network. At 28 days post-lesion, PDGFR-β expression was preferentially localized intraparenchymally over the extravascular areas in the lesion core, with some expression associated with the vasculature (Figures 5C,D). In addition, double-labeling for PDGFR-β and NG2 revealed that the extravascular network mainly composed of PDGFR-β-positive processes were devoid of NG2 immunoreactivity (Figures 5E–G). Quantitative temporal analysis revealed a progressive increase in the extravascular area occupied by PDGFR-β expression in the lesion core at 7–28 days after 3-NP injection (Figure 5H). In contrast, the size of the lesion core, which was assessed by measuring the maximum horizontal distance of the GFAP-negative striatal area, gradually decreased over time until at least 28 days (Figure 5I), indicating that the extravascular PDGFR-β-positive area and the lesion core revealed an inverse temporal pattern.

Ultrastructural features of PDGFR-β-positive cells in the lesion core, as shown in Figure 4, indicated that they seemed to produce collagen fibrils. Thus, we investigated the spatiotemporal relationship between PDGFR-β-positive cells and collagen in the lesioned striatum. In the control striatum, PDGFR-β was localized to some blood vessels, within which immunoreactivity for collagen IV presented as fine fibrillar profiles (Figures 5J,K). At 7 days post-lesion, intense PDGFR-β immunoreactivity was seen in most blood vessels, and was spatially correlated with a substantial increase in the amount of collagen IV as compared with the controls (Figures 5L,M). At 28 days post-lesion, collagen IV and PDGFR-β shared overlapping expression in both vascular profiles and extravascular areas (Figures 5N,O). This finding was further supported by the intensity profiles of PDGFR-β-positive and collagen IV-positive signals within the vascular and extravascular areas, which revealed similar localization (Figure 5P).

### PDGFR-β-Positive Cells Undergo Morphological Changes in the Lesion Core in the Chronic Phase After 3-NP Injection

**Figure 6:**
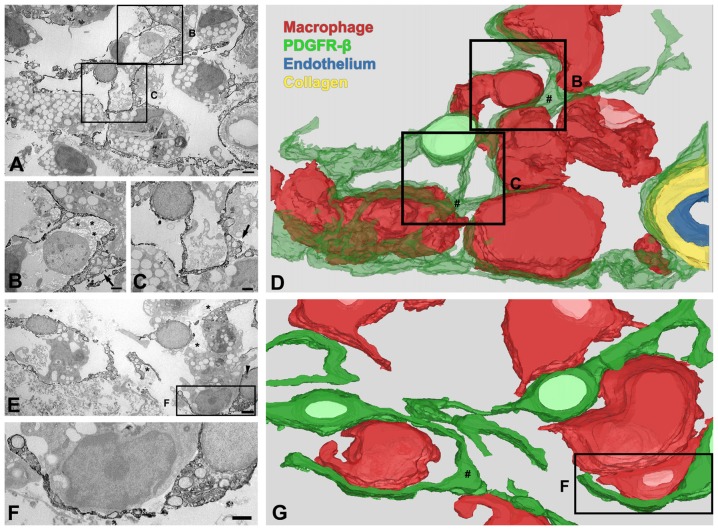
Three-dimensional (3D) images of PDGFR-β-positive cells in the lesion core at day 14 after 3-NP injection. **(A–D)** 3D reconstruction **(D)** of one PDGFR-β-positive cell is prepared from a series of 27 serial ultrathin sections (90-nm-thick), one of which is shown in **(A–C)**. A PDGFR-β-positive cell has a soma located at a distance from the vasculature and extends highly branched cytoplasmic processes, which frequently have close apposition with microglia/macrophages with amoeboid morphology, with little to no intervening space. **(E–G)** Another example of 3D reconstruction **(G)** of three PDGFR-β-positive cells, prepared from a series of 26 consecutive sections, one of which is shown in **(E,F)**. The three cells extend highly branched cytoplasmic processes that are frequently in close apposition or even interwoven with each other, forming a network. **(B,C,F)** Higher magnification images of the boxed areas in **(A,E)**, respectively. PDGFR-β-positive cells have focal cytoplasmic enlargements (arrows in **B,C,E**; # in **D,G**), from which distal branches arise. Note that their somata and processes are closely associated with collagen fibrils (asterisks in **B,E**). The arrowhead in **(E)** denotes a nucleolus. Scale bars = 2 μm for **(A,E)**; 1 μm for **(B,C,F)**.

**Figure 7:**
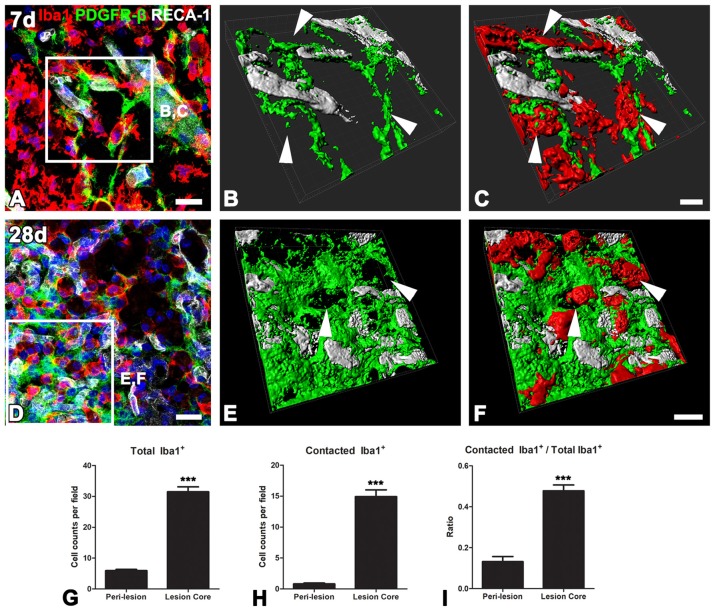
The close relationship between PDGFR-β-positive cells and activated microglia/macrophages in lesioned striatum. **(A–F)** Triple labeling for PDGFR-β, Iba1 and RECA-1 at days 7 **(A–C)** and 28 **(D–E)** post-lesion, showing that activated microglia/macrophages are in close proximity to a PDGFR-β-positive cell with highly branched processes. **(B,C,E,F)** Three-dimensional images with confocal microscopy of the boxed areas in **(A,E)**, respectively. White arrows in **(B,C,E,D)** denote the close contact points between both cells. **(G–I)** The numbers of total microglia/macrophages **(G)** and microglia/macrophages contacting PDGFR-β-positive cells **(H)** and the proportion of contacting microglia/macrophages **(I)** in the lesion core and peri-lesional area on day 7 post-lesion. Note that the numbers of both populations of microglia/macrophages and their proportion are significantly higher in the lesion core than in the peri-lesional area. The data are expressed as the mean ± SEM. ***P < 0.001 vs. the peri-lesional area data. Scale bars = 20 μm for **(A,D)**; 10v μm for **(B,C,E,F)**.

Ultrastructural investigation of PDGFR-β-positive cells on day 14 post-lesion revealed that their somata were located at a distance from the vasculature, although contact was maintained with the vasculature by some elongated processes (Figure 6A). These cells had euchromatic nuclei and conspicuous nucleoli and their somata and processes were closely associated with collagen fibrils (Figures 6A–C,E,F). In addition, they had highly branched cytoplasmic processes, which frequently had close apposition with microglia/macrophages, with multiple close contact points (Figures 6A–C,E,F). Despite this close apposition, no membranous specialization was found between them. Indeed, the close relationship between PDGFR-β-positive cells and activated microglia/macrophages in the lesion core could be clearly observed in three-dimensional confocal microscopy images (Figures 7A–F). In particular, microglia/macrophages with amoeboid morphology were observed in close proximity to PDGFR-β-positive cells, whose highly branched processes formed a cellular network among brain macrophages in the lesion core at 28 days post-lesion (Figures 7D–F). We quantified the numbers of total microglia/macrophages and microglia/macrophages contacting PDGFR-β-positive cells in the lesion core and peri-lesional area at day 7 post-lesion. As shown in Figures 7G,H, the numbers of both populations of microglia/macrophages were significantly higher in the lesion core than in the peri-lesional area. In particular, 47.82% of total microglia/macrophages were in close apposition to PDGFR-β-positive cells in the lesion core, as compared with only 13.2% in the peri-lesional area (Figure 7I).

We further examined the detailed morphology of PDGFR-β-positive cells and their spatial relationship using 3D reconstruction from a stack of serial ultrathin sections with a total thickness of 2.43 μm. As shown in Figures 6D,G, the cytoplasmic processes of PDGFR-β-positive cells were slender, even at their emergence from the cell body, and they had focal enlargements of cytoplasm containing abundant rER and mitochondria, from which thin branches often arose. In addition, highly branched cytoplasmic processes of PDGFR-β-positive cells were frequently in close apposition or even interwoven with each other, forming a network (Figure 6G).

### PDGFR-β-Positive Cells Express Nestin and Vimentin in the Core of Lesions Caused by 3-NP Treatment

**Figure 8:**
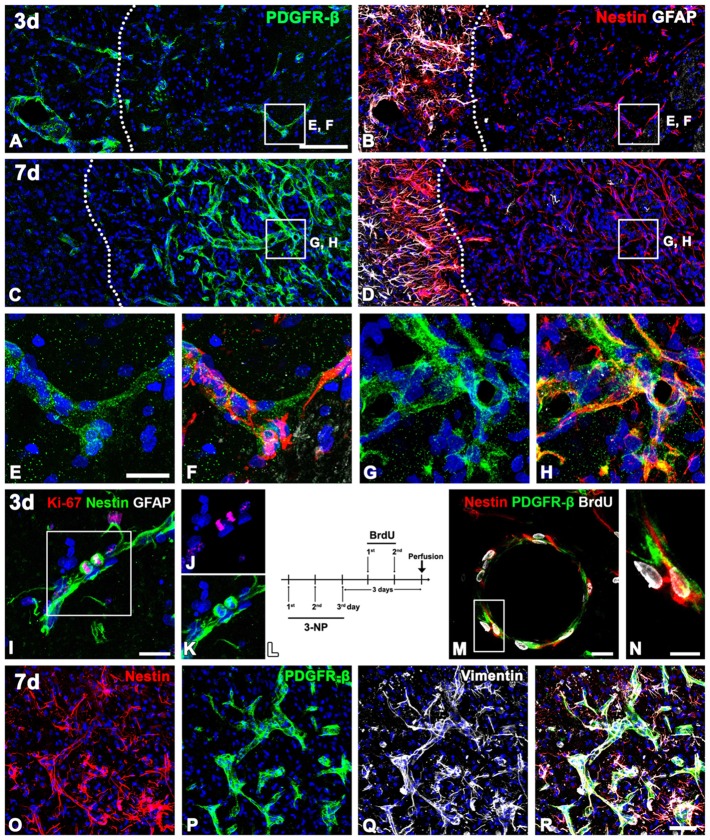
The spatiotemporal relationship of PDGFR-β and two intermediate filament proteins nestin and vimentin in the lesioned striatum at days 3 and 7 after 3-NP injection. **(A,B,E,F)** Triple-labeling for PDGFR-β, GFAP and nestin at day 3 post-lesion showing that vascular profiles expressing PDGFR-β are positive for nestin in the lesion core (right side of the broken line), which is devoid of GFAP immunoreactivity. Note that nestin-positive cells in the peri-lesional area are astrocytes. **(C,D,G,H)** At 7 days, distribution of PDGFR-β in the vascular profiles overlaps well with that of nestin in the lesion core (right side of the broken line). **(E–H)** Higher magnification images of the boxed areas in **(A–D)**, respectively. **(I–K)** Triple-labeling for nestin, Ki-67 and GFAP. Note that a pair of nestin-positive cells that appear to be daughter cells are positive for Ki-67. **(L)** 5-Bromo-2-deoxyuridine (BrdU) injection protocol. **(M,N)** Triple-labeling for nestin, BrdU and GFAP showing that PDGFR-β/nestin double-positive cells within vascular profiles exhibit BrdU-labeled cell nuclei. The boxed area in **(M)** is enlarged in **(N)**. **(O–R)** Triple labeling for PDGFR-β, nestin and vimentin showing that nearly all of PDGFR-β/nestin double-positive cells co-express vimentin. Cell nuclei are stained with DAPI. Scale bars = 100 μm for **(A–D)**; 20 μm for **(E–H,I,J–M)**; 10 μm for **(N)**; 50 μm for **(O–R)**.

The spatiotemporal distribution and morphology of PDGFR-β-positive cells in the lesion core were similar to those of nestin-positive cells, which were exclusively associated with vessels in the ischemic core of stroke-lesioned rats (Shin et al., 2013). Thus, to clarify whether PDGFR-β-positive cells express nestin, triple-labeling for PDGFR-β, nestin and GFAP was performed. In the control striatum, PDGFR-β-positive vascular profiles were devoid of significant immunoreactivities for nestin and GFAP (data not shown). At day 3 post-lesion, nestin and PDGFR-β expression overlapped in the lesion core, in which they were usually colocalized in vascular profiles (Figures 8A,B,E,F). In the peri-lesional area, however, nestin was colocalized with GFAP-positive reactive astrocytes (Figures 8A,B). On day 7, these labeling patterns were similar to those observed at day 3, but with a notable increase in the number of PDGFR-β/nestin double-positive vessels (Figures 8C,D,G,H). Triple-labeling for nestin, GFAP and the proliferation marker Ki-67 revealed that Ki-67-labeled nuclei were frequently present in nestin-positive vascular profiles (Figures 8I–K), and further, PDGFR-β/nestin double-positive cells within vascular profiles exhibited BrdU-labeled cell nuclei (Figures 8L–N), indicating that these were proliferating cells. In addition, nearly all PDGFR-β/nestin double-positive cells co-expressed vimentin, an intermediate filament protein (Figures 8O–R).

**Figure 9:**
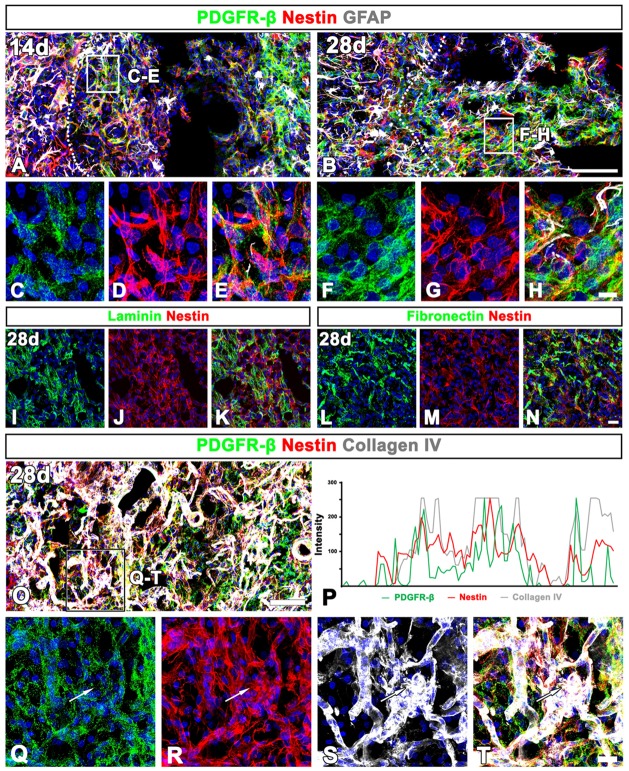
Characterization of extravascular PDGFR-β-positive cells in the lesioned striatum at days 14 and 28 after 3-NP injection. **(A–H)** Triple labeling for PDGFR-β, GFAP and nestin at day 14 **(A,C–E)** and 28 **(B,F–H)** post-lesion showing that the distribution of PDGFR-β overlaps well with that of nestin. Note that PDGFR-β-positive processes show finer intertwined profiles, while nestin staining reveals distinct fibrous structures. **(C–H)** Higher magnification images of the boxed areas in **(A,B)**, respectively. **(I–N)** Double labeling for nestin and either laminin **(I–K)** or fibronectin **(L–N)**, showing that nestin is colocalized with two stromal cell markers. **(O,Q–T)** Triple labeling for PDGFR-β, nestin and collagen IV showing that expression of the three proteins generally overlap in the lesion core. The boxed area in **(O)** is enlarged in **(Q–T)**. **(P)** Histogram of the intensity profiles of PDGFR-β, nestin and collagen IV along the indicated area (white arrows in **Q–S**) showing that the three signals share overlapping profiles. Cell nuclei are stained with DAPI. Scale bars = 100 μm for **(A,B,O)**; 20 μm for **(I–N„Q–T)**; 10 μm for **(C–H)**.

At day 14 post-lesion, the distribution of nestin still overlapped well with PDGFR-β in the lesion core, but the two proteins had somewhat different expression patterns; fine, intertwined PDGFR-β-positive processes appeared to form a network in vascular and extravascular areas, while nestin staining revealed distinct fibrous structures in these areas (Figures 9A,C–E). At this time point, sporadic astroglial processes were observed in the lesion core, but PDGFR-β and GFAP did not overlap. These localization patterns of PDGFR-β and nestin became more prominent at 28 days (Figures 9B,F–H). In addition, nestin colocalized with two stromal cell markers, laminin (Figures 9I–K) and the fibroblast marker fibronectin (Figures 9L–N) in the lesion core.

Next, we clarified whether the PDGFR-β/nestin double-positive cells were closely associated with collagen. Triple-labeling for PDGFR-β, nestin and collagen IV revealed that the three proteins generally overlapped throughout the lesion core at 28 days (Figures 9O,Q–T). This observation was further confirmed by their intensity profiles along the indicated area, which showed overall spatial correlation (Figure 9P).

**Figure 10:**
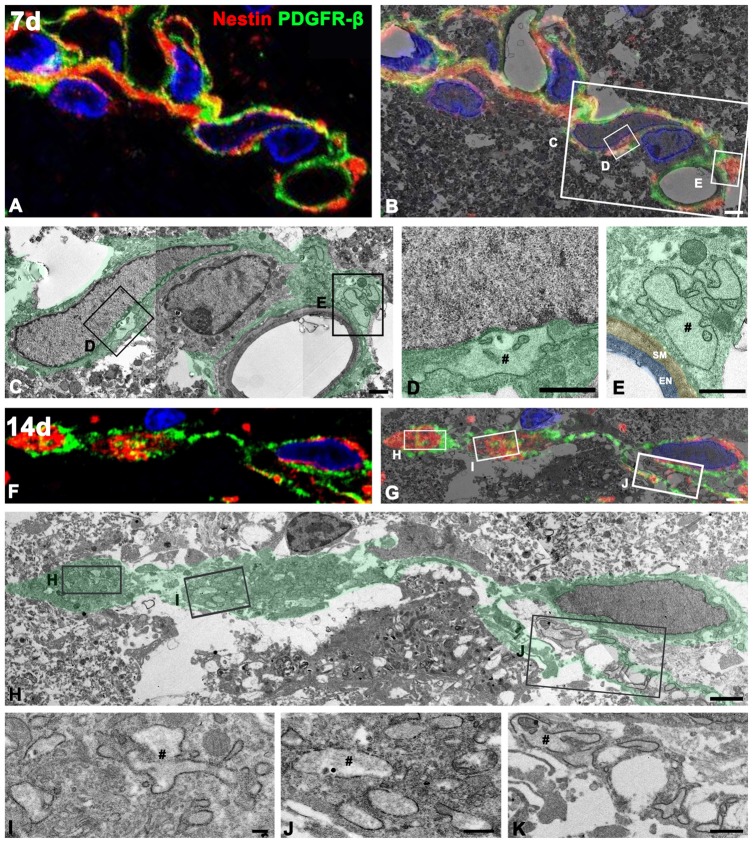
Ultrastructural characterization of PDGFR-β/nestin double-positive cells in the lesion core. Confocal microscopic image of a semi-thin section double-labeled with PDGFR-β and nestin **(A,F)**, the corresponding transmission electron microscopic image **(C,H)**, and the overlay image of confocal microscopic data and the corresponding electron microscopic image **(B,G)** obtained from days 7 **(A–E)** and 14 **(F–K)** post-lesion. **(D,E,L–K)** Higher-magnification views of the boxed areas in **(B,C,G,H)**, respectively. PDGFR-β/nestin double-positive cells have euchromatic nuclei and rER with dilated cisternae (#). Note that at 7 days, the cytoplasmic processes of these cells are still found extending around the vascular wall on the abluminal side of a smooth muscle (SM) cell and endothelial cells (EN), while at 14 days, they exhibit slender cytoplasmic processes branching out into the extravascular spaces. Cell nuclei are stained with DAPI. Scale bars = 2 μm for **(A,B)**, and **(F–H)**; 1 μm for **(C–E,K)**; 0.5 μm for **(J)**; 0.2 μm for **(I)**.

To define more precisely whether PDGFR-β-positive cells indeed express nestin, we used a correlative light- and electron-microscopic approach. Semi-thin sections double-labeled with PDGFR-β and nestin were first observed using confocal microscopy, which clearly revealed that the two proteins had nearly coinciding distributions in the lesion core at days 7 (Figure 10A) and 14 (Figure 10F) post-lesion. The same semi-thin sections were subsequently processed further for examination using electron microscopy (Figures 10B,G). Overlaying the confocal microscopy and transmission electron microscopy data confirmed that the ultrastructure of PDGFR-β/nestin double-positive cells was in accordance with the PDGFR-β-positive cells as described above; these cells had nuclei with predominant euchromatin and prominent dilatation of the rER cisternae could be seen (Figures 10C–E,H–K). At 7 days post-lesion, cytoplasmic processes were frequently found extending around the vascular wall (Figures 10C,E), while at 14 days, PDGFR-β/nestin double-positive cells exhibited slender cytoplasmic processes that branched out into the extravascular spaces (Figure 10H).

## Discussion

**Figure 11:**
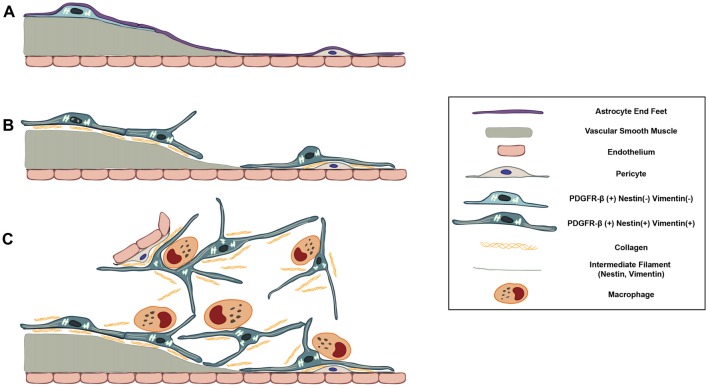
Schematic representation of dynamic PDGFR-β-positive cells in the lesion core in striata treated with 3-NP. **(A)** In the saline-treated control striatum, PDGFR-β-positive cells with thin processes are associated with larger caliber vessels having one or two layers of smooth muscle cells. These cells are located outside the smooth muscle cells and are surrounded by the glia limitans of astroglial processes. **(B)** At 3–7 days post-lesion, PDGFR-β-positive cells are highly proliferative and express nestin and vimentin. They have large euchromatic nuclei and dilated cisternae of rER, indicating the presence of active collagen synthesis. They also migrated to the adjacent microvascular wall and wrap around or directly abut the abluminal surface of endothelial cells or pericytes, despite the ongoing association with the vasculature. **(C)** At 14–28 days, the lesion core is progressively infiltrated by migrating PDGFR-β-positive cells expressing nestin and vimentin. They have highly branched cytoplasmic processes that are frequently in close apposition or are even interwoven with each other, forming a network. In addition, PDGFR-β-positive somata and processes have close apposition with brain macrophages, nearly always at the branch points of distal processes.

In the present study, we demonstrate the possibility that PDGFR-β-positive cells, localized in the perivascular adventitia, are responsible for fibrotic scar formation in the striata of rats subjected to 3-NP intoxication (Figure 11). These data provide novel insight into scar-forming perivascular fibroblasts after brain insults.

### PDGFR-β-Positive Cells Correspond to Typical Perivascular Fibroblasts in the Control Striatum and Could Contribute to Fibrotic Scar Formation in the Lesioned Striatum

In the control striatum, vessel-associated PDGFR-β-positive cells fit into the typical morphological features of perivascular fibroblasts (Peters et al., 1991), in that they were almost exclusively localized to larger caliber vessels, including arterioles and venules (25.5% of all vessels), and had flattened nuclei and thin cytoplasm, with long, slender cytoplasmic processes. PDGFR-β expression was increased and expanded to almost all vessels (99.6% of all vessels), including microvessels, at 7 days after 3-NP injection. In addition, PDGFR-β-positive cells underwent marked morphological changes, distinct from these cells in the controls, and exhibited larger euchromatic nuclei, conspicuous nucleoli and well-developed rERs with prominent cisternal dilation, implying that protein synthesis was actively proceeding in these cells. In addition, PDGFR-β and collagen IV, which are major extracellular matrix components in CNS fibrotic scars, with the latter also being the principle collagen type in the neurovascular basal lamina (Timpl and Brown, 1996; Klapka and Müller, 2006), shared overlapping expression in both vascular profiles and the extravascular area. Moreover, PDGFR-β-positive cells had membrane-bound granules containing filamentous materials, probably indicating the process of exportation of these materials into the extracellular space (Figure 4H). These morphological changes in PDGFR-β-positive cells are common features of fibroblasts undergoing active collagen synthesis.

PDGFR-β expression increased progressively in the extravascular areas during the chronic phase (days 14–28 post-lesion), although it was still present within vascular profiles. These PDGFR-β-positive cells had somata located at a distance from the vasculature, and their highly ramified, slender processes overlapped with those from other cells, thus forming a plexus of processes in the lesion core, which could be clearly seen in 3D analysis. In addition, these PDGFR-β -positive somata and processes were surrounded by many collagen fibrils. These data suggest that PDGFR-β-positive fibroblasts that originate in the vascular adventitia migrate into the injured parenchyma and contribute to fibrotic scar formation after acute brain insults. The possibility that parenchymal PDGFR-β-positive cells that co-labeled for NG2 are related to the increased PDGFR-β expression within the vascular and extravascular areas in the lesion core. However, our data clearly revealed that these parenchymal PDGFR-β/NG2 double-labeled cells were still detected in the extravascular area within the lesion core (Figure 3F). In addition, an extravascular network composed of PDGFR-β-positive processes, which we suggest as being responsible for fibrotic scar formation, were devoid of NG2 immunoreactivity (Figures 5E–G). Thus, these data suggest that extravascular PDGFR-β cells in the lesion core could be derived from vessel-associated PDGFR-β-positive cells, rather than from parenchymal PDGFR-β-positive cells.

The identity of the cells responsible for the fibrotic scars after CNS insults has long been debated. Pericytes or perivascular mesenchymal cells have recently been considered to be the source of fibrosis (Humphreys et al., 2010; Kramann et al., 2015; Di Carlo and Peduto, 2018). In agreement with this, Goritz et al. (2011), using a genetic lineage tracing technique, demonstrated that a subtype of pericytes, GLAST-positive type A pericytes, is responsible for fibrotic scar formation after spinal cord injury. These cells reside in the perivascular space in the uninjured state, but proliferate and migrate to the vasculature to produce fibrotic extracellular matrix components after injury. Similarly, Col1α1-positive perivascular fibroblasts were shown to be responsible for fibrotic scar formation after contusive spinal cord injury (Soderblom et al., 2013). Furthermore, Col1α1-positive cells were mostly localized in arterioles and venules in the uninjured spinal cord, which is similar to the location of PDGFR-β-positive adventitial fibroblasts in large-caliber vessels in the control striatum. It is therefore highly likely that these scar-forming cells, Col1α1-positive cells, GLAST-positive cells and PDGFR-β-positive fibroblasts are indeed the same type of perivascular adventitial fibroblasts presented in our study.

PDGFR-β-positive cells in the control and lesioned striata do not fit the classical definition of pericytes. Whereas pericytes have processes that extend around the capillary wall and are completely bounded by a basal lamina (Krueger and Bechmann, 2010; Attwell et al., 2016), PDGFR-β-positive cells in our study were found adluminal to smooth muscles in large-diameter vessels, but not in pericytes of capillaries in the control striatum. In addition, PDGFR-β-positive cells and processes are clearly segregated by the pericytes and the basal laminae of the pericytes that enveloped the endothelial cells in the lesion core (see Figures 4J–M). In particular, these cells seem more similar to perivascular fibroblasts, which are localized in vessels larger than capillaries in the brain (Peters et al., 1991). Interestingly, PDGFR-β-positive cells resemble leptomeningeal cells that form the tunica adventitia of larger vessels in that they have well-developed rERs indicative of active protein synthesis, and reside in the perivascular space (Zhang et al., 1990), suggesting that PDGFR-β-positive cells may be regarded as being derived from the leptomeninges.

PDGFR-β has been widely accepted as a marker for pericytes (Lindahl et al., 1997; Winkler et al., 2010; Armulik et al., 2011; Hartmann et al., 2015; Sweeney et al., 2016). However, it is not an exclusive or specific marker, because of its dynamic expression in other cell types. In the hippocampi of mice in which status epilepticus was induced, PDGFR-β has been shown to be expressed in NG2 glia and GFAP-positive astrocytes, as well as in pericytes (Kyyriäinen et al., 2017). PDGFR-β is also expressed in kidney mesangial cells and liver stellate cells, which act as fibroblasts in response to tissue damage (Andrae et al., 2008). Although not clearly distinguished from other perivascular cells, Fernández-Klett et al. (2013) also suggested that newly proliferated, PDGFR-β-expressing vascular stromal cells are the fibroblasts responsible for fibrotic scar-formation after cerebral ischemic stroke. Thus, our observations here provide ultrastructural evidence that vessel-associated PDGFR-β-positive cells represent perivascular fibroblasts, but not pericytes, in the control striatum and in the striatum subjected to 3-NP treatment. However, we cannot exclude the possibility that differences in the findings of our own and other studies could be due to the specific brain area studied, or species differences.

### Intermediate Filaments Nestin and Vimentin Are Induced in Activated PDGFR-β-Positive Fibroblasts

Interestingly, our data revealed that spatiotemporal expression of PDGFR-β and nestin exhibited overlapping expression patterns in the striatum of 3-NP-lesioned rats, although observation under light microscopy showed differential expression patterns of the two proteins: nestin, with long filamentous morphology, and PDGFR-β, distributed diffusely in the cytoplasm. The correlative light- and electron-microscopy technique confirmed that expression of the intermediate filament nestin is induced in PDGFR-β-positive perivascular fibroblasts in the lesion core. We previously demonstrated that nestin expression is induced in vasculature-associated cells, which appear to undergo dynamic structural changes in response to ischemic injury; however, their exact cellular identity had not been elucidated (Shin et al., 2013). The present study suggested that nestin-positive cells could be perivascular PDGFR-β-positive fibroblasts present in the adventitial layer. In addition to nestin, perivascular PDGFR-β-positive fibroblasts expressed the type III intermediate filament vimentin. Given that, unlike other types of intermediate filament proteins, nestin is unable to self-assemble, and requires other types of intermediate filament proteins, such as vimentin or desmin, to assemble into heterodimers (Eliasson et al., 1999; Wiese et al., 2004; Lowery et al., 2015), it is possible that vimentin, but not GFAP, forms a heterodimer with nestin in PDGFR-β-positive perivascular fibroblasts.

Intermediate filament proteins are well known for their dynamic roles in cytostructural remodeling (Leduc and Etienne-Manneville, 2015; Lowery et al., 2015). Nestin silencing in cultured podocytes leads to reduced cell process formation (Chen et al., 2006) and knockdown of nestin results in decreased migration and contractility in neural stem cells (Yan et al., 2016). In the present study, nestin was specifically expressed along the elongated processes of PDGFR-β-positive fibroblasts, both within the vasculature, in the early phase, and extending into the extravascular space, in the late phase. This may mean that PDGFR-β-positive perivascular fibroblasts require nestin to elongate their processes and migrate into the lesion core to form a fibrotic scar.

It should also be considered that nestin is expressed in non-neural cells with dynamic roles in various pathophysiological processes, including pathological fibrosis in the heart and kidneys (Lendahl et al., 1990; Wiese et al., 2004; Kishaba et al., 2010; Park et al., 2010; Calderone, 2012). A recent study showed induction of nestin expression in proliferative vascular smooth muscle cells and suggested that it plays an active role in vascular remodeling in pulmonary fibrosis (Saboor et al., 2016). Furthermore, collagen type I-expressing mesenchymal cells expressed nestin in pressure-overloaded ventricles to form fibrotic heart walls (Hertig et al., 2017). Taken together, our data raise the possibility that nestin expression in fibroblasts is a universal process in fibrotic scar formation after different types of injuries in any organ.

### Brain Macrophages Could Mediate the Migration of PDGFR-β-Positive Cells and Subsequent Fibrosis in the Lesion Core

In the lesion core, PDGFR-β-positive cells frequently made close contact with brain macrophages with amoeboid morphology, although no specific specialization connecting the two contiguous membranes was observed (Figure 5). Moreover, whenever PDGFR-β-positive processes extended distal processes, they showed close plasmalemmal apposition with brain macrophages, and had local cytoplasmic swelling, and contained prominent rERs and mitochondria. Interestingly, the presence of perivascular fibroblast/macrophage networks has been reported in the subependymal layer and richly vascularized zone of the hypothalamic nuclei, suggesting that connective tissue cells (macrophages and fibroblasts) may intervene actively in mechanisms of tissue plasticity and in the capability of these tissues to initiate and control cell proliferation and differentiation (Mercier et al., 2002, 2003). Thus, close cell-to-cell contacts between brain macrophages and fibroblast-like cells expressing PDGFR-β in the lesion core may have functional relevance.

Accumulating evidence has demonstrated that macrophages could promote tissue fibrosis, and depletion of macrophages lowers the amount of scar tissue deposited in the wounds, indicating that macrophages play a major role in wound healing, and in the progression of fibrosis (Leibovich and Ross, 1975; Duffield et al., 2005; Lucas et al., 2010). In addition, macrophages appear to progress from pro-inflammatory (M1) to anti-inflammatory and remodeling (M2) phenotypes during wound healing, and chronic activation of M2 macrophages leads to exacerbation of fibrosis by production of pro-fibrotic factors, such as transforming growth factor-beta (TGF-β) and galactin-3 (Vernon et al., 2010; Braga et al., 2015; White and Gomer, 2015; Zhu et al., 2016). Similar results were found in the injured spinal cord; deletion of hematogenous macrophages reduces fibroblast accumulation, suggesting that macrophages recruit perivascular fibroblasts to the injury site through cytokine expression (Zhu et al., 2015). The early-infiltrating hematogenous monocytes contribute to fibrotic scar formation, as evidenced by decreased levels of fibronectin and collagen IV, without altering glial scarring (Jeong et al., 2017). Furthermore, several studies have demonstrated a close spatial and temporal relationship between macrophages and fibroblasts in the wound healing process in the kidneys and skin: macrophages induce fibrosis through the recruitment, proliferation and activation of fibroblasts (Nishida et al., 2008; Nikolic-Paterson et al., 2014; Knipper et al., 2015; Zhu et al., 2017). Based on these prior observations, our data reinforce the concept that brain macrophages contribute to fibrotic scar formation after CNS injuries, and provide morphological evidence that these cells could provide important environmental cues for the migration and branching of PDGFR-β-positive fibroblasts in the lesion core. However, it is also possible that close plasmalemmal appositions of PDGFR-β-positive fibroblasts and brain macrophages are in physical contact simply because brain macrophages comprise the vast majority of cells in the lesion core.

In conclusion, using a rat model of acute brain injury, our data have shown the following: (1) PDGFR-β-positive perivascular adventitial fibroblasts could be discriminated from endothelial cells, pericytes, and smooth muscle cells. (2) The structural dynamics of PDGFR-β-positive cells correlate with progression of fibrotic scar formation: they formed a plexus of processes throughout the lesion core and showed a close spatial and temporal relationship with collagen IV. (3) PDGFR-β-positive fibroblasts express the intermediate filament proteins nestin and vimentin, the induction of which may account for dynamic structural changes after brain insults. (4) Their ultrastructural morphology and spatial correlation with brain macrophages were elaborated by 3D reconstruction of microscopic images in our study. Our results provide novel evidence of the morphology and potential roles of PDGFR-β-positive perivascular fibroblasts in the development of fibrotic scar formation after CNS insults.

## Author Contributions

All authors have contributed significantly to the research and the article preparation: T-RR contributed to the treatment of animals, immunoblot assays, immunohistochemistry, immunoelectron microscopy, serial section-based 3-dimensional reconstruction and quantitative analysis. J-HC and XJ contributed to the treatment of animals and immunohistochemistry. HK worked on the electron microscopy. M-YL worked on the design of the study, data analysis and final manuscript preparation.

## Conflict of Interest Statement

The authors declare that the research was conducted in the absence of any commercial or financial relationships that could be construed as a potential conflict of interest.

## Abbreviations

3-NP: 3-nitropropionic acid
ANOVA: one-way analysis of variance
CNS: central nervous system
DARPP-32: 32 kDa dopamine- and cyclic AMP-regulated phosphoprotein
GFAP: glial fibrillary acidic protein
PDGFR-β: platelet-derived growth factor receptor beta
rER: rough endoplasmic reticulum.

## References

[B1] Andrae J. Gallini R. Betsholtz C. (2008). Role of platelet-derived growth factors in physiology and medicine. Genes Dev. 22, 1276–1312. 10.1101/gad.1653708 18483217 PMC2732412

[B2] Armulik A. Genové G. Betsholtz C. (2011). Pericytes: developmental, physiological, and pathological perspectives, problems, and promises. Dev. Cell 21, 193–215. 10.1016/j.devcel.2011.07.001 21839917

[B3] Attwell D. Mishra A. Hall C. N. O’Farrell F. M. Dalkara T. (2016). What is a pericyte? J. Cereb. Blood Flow Metab. 36, 451–455. 10.1177/0271678X15610340 26661200 PMC4759679

[B4] Braga T. T. Agudelo J. S. Camara N. O. (2015). Macrophages during the fibrotic process: M2 as friend and foe. Front. Immunol. 6:602. 10.3389/fimmu.2015.00602 26635814 PMC4658431

[B5] Brouillet E. Jacquard C. Bizat N. Blum D. (2005). 3-Nitropropionic acid: a mitochondrial toxin to uncover physiopathological mechanisms underlying striatal degeneration in Huntington’s disease. J. Neurochem. 95, 1521–1540. 10.1111/j.1471-4159.2005.03515.x 16300642

[B6] Calderone A. (2012). Nestin+ cells and healing the infarcted heart. Am. J. Physiol. Heart Circ. Physiol. 302, H1–H9. 10.1152/ajpheart.00716.2011 22003051

[B7] Cha J.-H. Wee H.-J. Seo J. H. Ahn B.-J. Park J.-H. Yang J.-M.. (2014). AKAP12 mediates barrier functions of fibrotic scars during CNS repair. PLoS One 9:e94695. 10.1371/journal.pone.0094695 24760034 PMC3997571

[B8] Chen J. Boyle S. Zhao M. Su W. Takahashi K. Davis L.. (2006). Differential expression of the intermediate filament protein nestin during renal development and its localization in adult podocytes. J. Am. Soc. Nephrol. 17, 1283–1291. 10.1681/asn.2005101032 16571784

[B9] Choi J.-H. Riew T.-R. Kim H. L. Jin X. Lee M.-Y. (2017). Desmin expression profile in reactive astrocytes in the 3-nitropropionic acid-lesioned striatum of rat: characterization and comparison with glial fibrillary acidic protein and nestin. Acta Histochem. 119, 795–803. 10.1016/j.acthis.2017.10.003 29054283

[B10] Di Carlo S. E. Peduto L. (2018). The perivascular origin of pathological fibroblasts. J. Clin. Invest. 128, 54–63. 10.1172/jci93558 29293094 PMC5749494

[B11] Duffield J. S. Forbes S. J. Constandinou C. M. Clay S. Partolina M. Vuthoori S.. (2005). Selective depletion of macrophages reveals distinct, opposing roles during liver injury and repair. J. Clin. Invest. 115, 56–65. 10.1172/jci200522675 15630444 PMC539199

[B12] Eliasson C. Sahlgren C. Berthold C. H. Stakeberg J. Celis J. E. Betsholtz C.. (1999). Intermediate filament protein partnership in astrocytes. J. Biol. Chem. 274, 23996–24006. 10.1074/jbc.274.34.23996 10446168

[B13] Fernández-Klett F. Priller J. (2014). The fibrotic scar in neurological disorders. Brain Pathol. 24, 404–413. 10.1111/bpa.12162 24946078 PMC8029481

[B14] Fernández-Klett F. Potas J. R. Hilpert D. Blazej K. Radke J. Huck J.. (2013). Early loss of pericytes and perivascular stromal cell-induced scar formation after stroke. J. Cereb. Blood Flow Metab. 33, 428–439. 10.1038/jcbfm.2012.187 23250106 PMC3587816

[B15] Garbelli R. de Bock F. Medici V. Rousset M. C. Villani F. Boussadia B.. (2015). PDGFRβ^+^ cells in human and experimental neuro-vascular dysplasia and seizures. Neuroscience 306, 18–27. 10.1016/j.neuroscience.2015.07.090 26283024

[B16] Goritz C. Dias D. O. Tomilin N. Barbacid M. Shupliakov O. Frisen J. (2011). A pericyte origin of spinal cord scar tissue. Science 333, 238–242. 10.1126/science.1203165 21737741

[B17] Hamilton B. F. Gould D. H. (1987). Nature and distribution of brain lesions in rats intoxicated with 3-nitropropionic acid: a type of hypoxic (energy deficient) brain damage. Acta Neuropathol. 72, 286–297. 10.1007/bf00691103 3564909

[B18] Hartmann D. A. Underly R. G. Grant R. I. Watson A. N. Lindner V. Shih A. Y. (2015). Pericyte structure and distribution in the cerebral cortex revealed by high-resolution imaging of transgenic mice. Neurophotonics 2:041402. 10.1117/1.nph.2.4.041402 26158016 PMC4478963

[B19] Hertig V. Tardif K. Meus M. A. Duquette N. Villeneuve L. Toussaint F.. (2017). Nestin expression is upregulated in the fibrotic rat heart and is localized in collagen-expressing mesenchymal cells and interstitial CD31^+^- cells. PLoS One 12:e0176147. 10.1371/journal.pone.0176147 28448522 PMC5407835

[B20] Humphreys B. D. Lin S. L. Kobayashi A. Hudson T. E. Nowlin B. T. Bonventre J. V.. (2010). Fate tracing reveals the pericyte and not epithelial origin of myofibroblasts in kidney fibrosis. Am. J. Pathol. 176, 85–97. 10.2353/ajpath.2010.090517 20008127 PMC2797872

[B21] Jeong S. J. Cooper J. G. Ifergan I. McGuire T. L. Xu D. Hunter Z.. (2017). Intravenous immune-modifying nanoparticles as a therapy for spinal cord injury in mice. Neurobiol. Dis. 108, 73–82. 10.1016/j.nbd.2017.08.006 28823935 PMC5675775

[B22] Kim H. L. Lee M. Y. Shin Y. J. Song D. W. Park J. Chang B. S.. (2015). Increased expression of osteopontin in the degenerating striatum of rats treated with mitochondrial toxin 3-nitropropionic acid: a light and electron microscopy study. Acta Histochem. Cytochem. 48, 135–143. 10.1267/ahc.15010 26633905 PMC4652028

[B23] Kishaba Y. Matsubara D. Niki T. (2010). Heterogeneous expression of nestin in myofibroblasts of various human tissues. Pathol. Int. 60, 378–385. 10.1111/j.1440-1827.2010.02532.x 20518888

[B24] Klapka N. Müller H. W. (2006). Collagen matrix in spinal cord injury. J. Neurotrauma 23, 422–435. 10.1089/neu.2006.23.422 16629627

[B25] Knipper J. A. Willenborg S. Brinckmann J. Bloch W. Maaß T. Wagener R.. (2015). Interleukin-4 receptor α signaling in myeloid cells controls collagen fibril assembly in skin repair. Immunity 43, 803–816. 10.1016/j.immuni.2015.09.005 26474656 PMC4681399

[B26] Kramann R. Schneider R. K. DiRocco D. P. Machado F. Fleig S. Bondzie P. A.. (2015). Perivascular Gli1^+^ progenitors are key contributors to injury-induced organ fibrosis. Cell Stem Cell 16, 51–66. 10.1016/j.stem.2014.11.004 25465115 PMC4289444

[B27] Krueger M. Bechmann I. (2010). CNS pericytes: concepts, misconceptions, and a way out. Glia 58, 1–10. 10.1002/glia.20898 19533601

[B28] Kyyriäinen J. Ekolle Ndode-Ekane X. Pitkänen A. (2017). Dynamics of PDGFRβ expression in different cell types after brain injury. Glia 65, 322–341. 10.1002/glia.23094 27778377

[B29] Leduc C. Etienne-Manneville S. (2015). Intermediate filaments in cell migration and invasion: the unusual suspects. Curr. Opin. Cell Biol. 32, 102–112. 10.1016/j.ceb.2015.01.005 25660489

[B30] Leibovich S. J. Ross R. (1975). The role of the macrophage in wound repair. A study with hydrocortisone and antimacrophage serum. Am. J. Pathol. 78, 71–100. 1109560 PMC1915032

[B31] Lendahl U. Zimmerman L. B. McKay R. D. (1990). CNS stem cells express a new class of intermediate filament protein. Cell 60, 585–595. 10.1016/0092-8674(90)90662-x 1689217

[B32] Lindahl P. Johansson B. R. Levéen P. Betsholtz C. (1997). Pericyte loss and microaneurysm formation in PDGF-B-deficient mice. Science 277, 242–245. 10.1126/science.277.5323.242 9211853

[B33] Lowery J. Kuczmarski E. R. Herrmann H. Goldman R. D. (2015). Intermediate filaments play a pivotal role in regulating cell architecture and function. J. Biol. Chem. 290, 17145–17153. 10.1074/jbc.r115.640359 25957409 PMC4498054

[B34] Lucas T. Waisman A. Ranjan R. Roes J. Krieg T. Müller W.. (2010). Differential roles of macrophages in diverse phases of skin repair. J. Immunol. 184, 3964–3977. 10.4049/jimmunol.0903356 20176743

[B35] Mercier F. Kitasako J. T. Hatton G. I. (2002). Anatomy of the brain neurogenic zones revisited: fractones and the fibroblast/macrophage network. J. Comp. Neurol. 451, 170–188. 10.1002/cne.10342 12209835

[B36] Mercier F. Kitasako J. T. Hatton G. I. (2003). Fractones and other basal laminae in the hypothalamus. J. Comp. Neurol. 455, 324–340. 10.1002/cne.10496 12483685

[B37] Nakagomi T. Molnár Z. Nakano-Doi A. Taguchi A. Saino O. Kubo S.. (2011). Ischemia-induced neural stem/progenitor cells in the pia mater following cortical infarction. Stem Cells Dev. 20, 2037–2051. 10.1089/scd.2011.0279 21838536

[B38] Nakata M. Nakagomi T. Maeda M. Nakano-Doi A. Momota Y. Matsuyama T. (2017). Induction of perivascular neural stem cells and possible contribution to neurogenesis following transient brain ischemia/reperfusion injury. Transl. Stroke Res. 8, 131–143. 10.1007/s12975-016-0479-1 27352866

[B39] Nikolic-Paterson D. J. Wang S. Lan H. Y. (2014). Macrophages promote renal fibrosis through direct and indirect mechanisms. Kidney Int. Suppl. 4, 34–38. 10.1038/kisup.2014.7 26312148 PMC4536961

[B40] Nishida M. Okumura Y. Sato H. Hamaoka K. (2008). Delayed inhibition of p38 mitogen-activated protein kinase ameliorates renal fibrosis in obstructive nephropathy. Nephrol. Dial. Transplant 23, 2520–2524. 10.1093/ndt/gfn309 18515792

[B41] Park D. Xiang A. P. Mao F. F. Zhang L. Di C.-G. Liu X.-M.. (2010). Nestin is required for the proper self-renewal of neural stem cells. Stem Cells 28, 2162–2171. 10.1002/stem.541 20963821

[B42] Paxinos G. Watson C. (2006). The Rat Brain in Stereotaxic Coordinates. 6th Edn. New York, NY: Academic Press.

[B43] Peters A. Palay S. Webster H. (1991). The Fine Structure of the Nervous System: The Neurons and Supporting Cells. New York, NY: Oxford University Press.

[B44] Riew T.-R. Kim H. L. Choi J.-H. Jin X. Shin Y.-J. Lee M.-Y. (2017a). Progressive accumulation of autofluorescent granules in macrophages in rat striatum after systemic 3-nitropropionic acid: a correlative light- and electron-microscopic study. Histochem. Cell Biol. 148, 517–528. 10.1007/s00418-017-1589-x 28597061

[B45] Riew T.-R. Kim H. L. Jin X. Choi J.-H. Shin Y.-J. Kim J. S.. (2017b). Spatiotemporal expression of osteopontin in the striatum of rats subjected to the mitochondrial toxin 3-nitropropionic acid correlates with microcalcification. Sci. Rep. 7:45173. 10.1038/srep45173 28345671 PMC5366947

[B46] Saboor F. Reckmann A. N. Tomczyk C. U. Peters D. M. Weissmann N. Kaschtanow A.. (2016). Nestin-expressing vascular wall cells drive development of pulmonary hypertension. Eur. Respir. J. 47, 876–888. 10.1183/13993003.00574-2015 26699726 PMC5796529

[B47] Shin Y.-J. Kim H. L. Park J.-M. Cho J. M. Kim S. Y. Lee M.-Y. (2013). Characterization of nestin expression and vessel association in the ischemic core following focal cerebral ischemia in rats. Cell Tissue Res. 351, 383–395. 10.1007/s00441-012-1538-x 23250576

[B48] Soderblom C. Luo X. Blumenthal E. Bray E. Lyapichev K. Ramos J.. (2013). Perivascular fibroblasts form the fibrotic scar after contusive spinal cord injury. J. Neurosci. 33, 13882–13887. 10.1523/JNEUROSCI.2524-13.2013 23966707 PMC3755723

[B49] Sweeney M. D. Ayyadurai S. Zlokovic B. V. (2016). Pericytes of the neurovascular unit: key functions and signaling pathways. Nat. Neurosci. 19, 771–783. 10.1038/nn.4288 27227366 PMC5745011

[B50] Timpl R. Brown J. C. (1996). Supramolecular assembly of basement membranes. Bioessays 18, 123–132. 10.1002/bies.950180208 8851045

[B51] Vernon M. A. Mylonas K. J. Hughes J. (2010). Macrophages and renal fibrosis. Semin. Nephrol. 30, 302–317. 10.1016/j.semnephrol.2010.03.004 20620674

[B52] White M. J. Gomer R. H. (2015). Trypsin, tryptase, and thrombin polarize macrophages towards a pro-fibrotic M2a phenotype. PLoS One 10:e0138748. 10.1371/journal.pone.0138748 26407067 PMC4583378

[B53] Wiese C. Rolletschek A. Kania G. Blyszczuk P. Tarasov K. V. Tarasova Y.. (2004). Nestin expression—a property of multi-lineage progenitor cells? Cell. Mol. Life Sci. 61, 2510–2522. 10.1007/s00018-004-4144-6 15526158 PMC11924557

[B54] Winkler E. A. Bell R. D. Zlokovic B. V. (2010). Pericyte-specific expression of PDGF β receptor in mouse models with normal and deficient PDGF β receptor signaling. Mol. Neurodegener. 5:32. 10.1186/1750-1326-5-32 20738866 PMC2936891

[B55] Yan S. Li P. Wang Y. Yu W. Qin A. Liu M.. (2016). Nestin regulates neural stem cell migration via controlling the cell contractility. Int. J. Biochem. Cell Biol. 78, 349–360. 10.1016/j.biocel.2016.07.034 27477313

[B56] Yoshioka N. Hisanaga S. Kawano H. (2010). Suppression of fibrotic scar formation promotes axonal regeneration without disturbing blood-brain barrier repair and withdrawal of leukocytes after traumatic brain injury. J. Comp. Neurol. 518, 3867–3881. 10.1002/cne.22431 20653039

[B57] Zhang E. T. Inman C. B. Weller R. O. (1990). Interrelationships of the pia mater and the perivascular (Virchow-Robin) spaces in the human cerebrum. J. Anat. 170, 111–123. 2254158 PMC1257067

[B59] Zhu Z. Ding J. Ma Z. Iwashina T. Tredget E. E. (2016). Systemic depletion of macrophages in the subacute phase of wound healing reduces hypertrophic scar formation. Wound Repair Regen. 24, 644–656. 10.1111/wrr.12442 27169512

[B60] Zhu Z. Ding J. Ma Z. Iwashina T. Tredget E. E. (2017). Alternatively activated macrophages derived from THP-1 cells promote the fibrogenic activities of human dermal fibroblasts. Wound Repair Regen. 25, 377–388. 10.1111/wrr.12532 28370945

[B58] Zhu Y. Soderblom C. Krishnan V. Ashbaugh J. Bethea J. R. Lee J. K. (2015). Hematogenous macrophage depletion reduces the fibrotic scar and increases axonal growth after spinal cord injury. Neurobiol. Dis. 74, 114–125. 10.1016/j.nbd.2014.10.024 25461258 PMC4323620

